# Prevalence and factors associated with musculoskeletal pain among hospital cleaning staff

**DOI:** 10.1590/0034-7167-2023-0237

**Published:** 2024-12-16

**Authors:** Emanuelli Mancio Ferreira da Luz, Oclaris Lopes Munhoz, Patrícia Bitencourt Toscani Greco, Bruna Xavier Morais, Silviamar Camponogara, Tânia Solange Bosi de Souza Magnago

**Affiliations:** I Universidade Federal do Rio Grande. Rio Grande, Rio Grande do Sul, Brazil; II Universidade Federal de Santa Maria. Santa Maria, Rio Grande do Sul, Brazil

**Keywords:** Saúde do Trabalhador, Dor Musculoesquelética, Transtornos Traumáticos Cumulativos, Serviço Hospitalar de Limpeza, Serviços Hospitalares., Salud Laboral, Dolor Musculoesquelético, Trastornos de Traumas Acumulados, Servicio de Limpieza en Hospital, Servicios Hospitalarios.

## Abstract

**Objetivos:**

verificar a prevalência e os fatores associados à dor musculoesquelética em trabalhadores do serviço hospitalar de limpeza.

**Métodos:**

estudo transversal, realizado com trabalhadores de limpeza de um hospital de ensino do Sul do Brasil. Utilizaram-se questionário com variáveis sociodemográficas, laborais e de saúde, o *Nordic Musculoskeletal Questionnaire* e o Diagrama de Corlett e Manenica. Realizou-se análise bivariada.

**Resultados:**

participaram 149 trabalhadores. Prevaleceu dor musculoesquelética na coluna lombar no último ano (65,8%) e últimos sete dias (42,3%). Constataram-se associações entre automedicação e dor na parte inferior das costas (p=0,020) e ombros (p=0,026); sedentarismo, oito horas de sono diárias e dor nos tornozelos (p=0,041) e pés (p=0,039); ex-tabagismo, uso de medicamento e dor nos punhos (p=0,015) e mãos (p=0,004).

**Conclusões:**

prevaleceram lombalgias associadas a hábitos de saúde e vida. Um programa de educação em saúde e recomendações de melhorias nos processos de trabalho podem minimizar a exposição à dor musculoesquelética.

## RESUMEN

**Objetivos:**

verificar la prevalencia y los factores asociados al dolor musculoesquelético en trabajadores del servicio hospitalario de limpieza.

**Métodos:**

estudio transversal, realizado con trabajadores de limpieza de un hospital de enseñanza en el sur de Brasil. Se utilizaron cuestionarios con variables sociodemográficas, laborales y de salud, el *Nordic Musculoskeletal Questionnaire* y el Diagrama de Corlett y Manenica. Se realizó análisis bivariado.

**Resultados:**

participaron 149 trabajadores. Prevaleció el dolor musculoesquelético en la columna lumbar en el último año (65,8%) y últimos siete días (42,3%). Se constataron asociaciones entre automedicación y dolor en la parte inferior de la espalda (p=0,020) y hombros (p=0,026); sedentarismo, ocho horas de sueño diarias y dolor en los tobillos (p=0,041) y pies (p=0,039); ex-tabaquismo, uso de medicamento y dolor en los puños (p=0,015) y manos (p=0,004).

**Conclusiones:**

prevaleció el dolor lumbar asociado a hábitos de salud y vida. Un programa de educación en salud y recomendaciones de mejorías en los procesos de trabajo pueden minimizar la exposición al dolor musculoesquelético.

## INTRODUCTION

Musculoskeletal pain (MSP) results from excessive use of the musculoskeletal system associated with insufficient recovery time. It can be expressed through self-reported symptoms such as pain (in bones, joints, muscles, or surrounding structures), fatigue, and paresthesia^(1)^. MSP is a significant physical health problem and one of the leading problems affecting workers in a wide range of occupational groups exposed to high physical demands and subject to constant movements^(2)^.

Cleaning staff in hospitals deals with dynamic and intense work demands^(3)^, which may overload the musculoskeletal system, causing physical exhaustion and resulting in MSP^(4)^. From this perspective, the exposure of cleaning workers to MSP may be associated with their work process, characterized by manual and repetitive tasks performed with little mechanical assistance and requiring physical force that is applied with an intense work pace^(5)^. Furthermore, cleaning tasks are characterized by non-ergonomic body postures associated with movements involving the frequent handling of loads and static muscle work in tasks such as pulling, pushing, standing, and walking^(3)^.

Even though such exposure is preventable, it overloads the workers’ musculoskeletal system, causing MSP. Therefore, it is the primary cause of different degrees of limitation and incapacity for work, causing the quality of services to drop. MSP accounts for the highest percentage of absenteeism, sometimes more prolonged and recurrent than absenteeism caused by other injuries^(6)^.

According to the Brazilian Social Security, musculoskeletal diseases account for the highest prevalence of conditions that require payment of sickness benefits in the last decade. In 2020, 150,361 urban and rural workers, and 303,399 in 2021 were granted such benefits due to diseases of the musculoskeletal system and connective tissues^(7)^. A prevalence of 70.1% is found among hospital cleaning workers, with 25.5% of symptoms considered intense and unbearable^(8)^.

Despite its relevance, few studies investigate cleaning workers’ exposure and musculoskeletal involvement, specifically those working in hospital settings. Additionally, there has been an increasing tendency of nurses to take on a role in hygiene and sanitation governance in hospital facilities to ensure adequate dimensioning for the safety of workers and clients and proper cleaning to minimize occupational infections and accidents^(9)^. Furthermore, the interface between the cleaning staff and nursing deserves to be highlighted due to the contemporaneity of the topic and the challenge imposed on nurses^(9)^, considering that investing in the health of cleaning workers and contributing to their visibility in institutions represents an innovation in nurses’ practice.

## OBJECTIVES

To verify the prevalence and factors associated with MSP among hospital cleaning workers.

## METHODS

### Ethical Aspects

The Institutional Review Board approved this study on August 14, 2018. The workers received guidance regarding this study’s objectives and signed two copies of a free informed consent form to confirm participation. All the ethical guidelines proposed by Resolution 466/2012, National Health Council, were complied with, ensuring voluntary participation, the anonymity of the participants’ identities, and confidentiality of their information.

### Study Design

STrengthening the Reporting of OBservational Studies in Epidemiology (STROBE)^(10)^ guided this study to ensure a clear and transparent redaction.

### Study Setting

This study was performed at a public university hospital in southern Brazil, where cleaning workers are hired by a third-party service provider. During the study period, 152 workers performed cleaning activities in the hospital’s facility and its 403 hospital beds.

### Sample selection criteria

The cleaning staff working in the facility for at least 30 days was included. This criterion was based on the assumption that 30 days would be the minimum for a cleaning worker to adapt to the organization and ward, having performed daily activities and being able to verify the presence of MSP in his/her work context. Workers on leave during data collection were excluded. Thus, a census sample of 149 (98%) participants answered the questionnaire. Losses (n=3; 2%) resulted from workers refusing to participate (n=2) and being absent on the date of data collection (n=1).

### Data Collection

Data were collected in July 2019 during regular work shifts in a private room after the manager authorized the participants to interrupt their work tasks without imposing any penalties. The primary author trained graduate students and students holding a scientific initiation scholarship to collect data (a pilot test was applied to check the application of the questionnaire and the data collection protocol). After training, those on the employee list and included in the work schedule were individually invited. Absent workers were contacted by telephone, and a new date was set for data collection.

A self-administered questionnaire was used to address sociodemographic information (i.e., sex, age group, education, marital status, and number of children), occupational characteristics (i.e., work shift, time in position, daily workload, leisure time, satisfaction with the number of people on the shift, whether the worker had another job, and had completed training on ergonomic risks), and health characteristics (i.e., smoking, alcohol consumption, medication use, a condition diagnosed by a physician, daily hours of sleep, physical exercise, and anthropometric measurements such as weight, height, abdominal and hip circumference, and Body Mass Index (BMI). The Brazilian version of the Nordic Musculoskeletal Questionnaire (NMQ)^(11)^ was applied to assess the MSP outcome. The Corlett and Manenica Diagram^(12)^, a map of body regions, was used as an auxiliary tool for the participants to report the anatomical regions when they experienced pain or bodily discomfort and its intensity^(12)^. Both the diagram and the NMQ are accessible free of charge^(11,12)^.

Workers who answered affirmatively to the NMQ question^(11)^: Have you at any time in the last year and last seven days had any pain or discomfort on (your neck, shoulders, elbows, wrists or hands, thoracic spine, lumbar spine, thighs, legs, knees, or ankles)? Additionally, workers could report whether they were impeded/absent from work in the last year^(11)^ and indicate the region of pain or musculoskeletal discomfort in the 25 regions represented in a human body figure divided into right and left, as well as the intensity of its occurrence^(12)^.

### Data Analysis

Data were entered into Epi Info^®^ software, version 6.4, with independent double entry. After checking for errors and inconsistencies, data were analyzed using the Statistical Package for the Social Sciences (SPSS®, SPSS Inc, Chicago), version 18.0, using descriptive and inferential statistics. Categorical variables were described using absolute (n) and relative frequencies (%). Quantitative variables were described using position and dispersion measures (mean or median, standard deviation, or interquartile range, respectively) depending on whether data were normally distributed (Kolmogorov-Smirnov test).

The cleaning staff was characterized according to sociodemographic, occupational, and health profiles using descriptive statistics. MSP in each anatomical region was a dichotomous variable (present or absent), described through absolute (N) and relative frequencies (%). Considering that the workers reported an average of 11 months on the job (59.7%), we considered MSP experienced in the seven days (dependent variable) in the bivariate analysis. The prevalence (P)^(13)^ of MSP was verified according to the formula:


$$P=\frac{\text{Nº of MSP reports in a given region and time}}{\text{population in the same region and time}}\times 100$$

MSP intensity in the last seven days, according to the anatomical region reported in the Corlett and Manenica Diagram, was classified from 0 (no pain) to 10 (intense/unbearable pain)^(12)^. Bivariate analyses were performed to identify associations between MSP in the last seven days and the independent variables (sociodemographic, work, lifestyle, and health). Pearson’s Chi-square and Fisher’s Exact tests were used, considering a 95% confidence interval (p<0.05). BMI was analyzed using the formula weight/(height)^2^ and classified according to international standards for adult assessment^(14)^.

## RESULTS

Most of the 149 (98%) participant cleaning workers were women (89.9%; n=134), aged from 49 to 62 (34.9%, n=52), with complete high school (36.2%; n=54), married or with a stable partner (55%; n=82), and with one to two children (53.7%). Occupational characterization indicated a prevalence of daytime workers (76.5%; n=114) with less than one year of work experience (59.7%; n=89), working six-hours a day (45%; n=67). The number of people on the work schedule was considered sufficient (53%; n=79); most did not report a second job (73.2%; n=109) and did not receive training on ergonomic risks (45%; n=67).

Regarding lifestyle and health, the highest percentage of workers reported having time for leisure (40.9%; N=61), not smoking (62.4%; n=93) or consuming alcohol (69.1%; n=103), and using at least one medication (47%; n=70); anti-inflammatory medications were the most frequent (48.3%; n=34). Regarding having a diagnosed disease, 40.3% (n=60) reported at least one medical condition. Musculoskeletal disorders were prevalent (tendinopathy, carpal tunnel syndrome, epicondylitis, bursitis, peripheral neuropathy, lumbar fissure, and cervical strain) (31.2%; n=46). The cleaning workers reported an average of 6.61 hours of sleep (±10.79), and most reported a sedentary lifestyle (64.4%; n=96). Regarding BMI, 38.9% (n=58) were overweight (BMI 25 to 29.9 kg/m^2^), with 36.2% (n=54) presenting obesity Classes I, II, and III. The waist/hip ratio showed an average of 0.90 cm (±0.06), a minimum of 0.63 cm, and a maximum of 1.10 cm.

Table 1 presents the prevalence of MSP reported in the last year and in the seven days before the interview according to the anatomical location.

**Table 1:** Distribution of musculoskeletal pain in the last 12 months and last seven days according to the anatomical location (Nordic Musculoskeletal Questionnaire) among hospital cleaning workers, Santa Maria, Rio Grande do Sul, Brazil, 2019 (N=149)

Anatomical location	Have you at any time in the last 12 months had trouble (pain, tingling, numbness) in:				Have you at any time during the last 12 months been prevented from doing your normal work because of the trouble in:				Have you had trouble at any time during the last 7 days in:			
	No		Yes		No		Yes		No		Yes	
	n	%	n	%	n	%	n	%	n	%	n	%
Neck	94	63.1	55	36.9	135	90.6	14	9.4	121	81.2	28	18.8
Shoulders	72	48.3	77	51.7	139	93.3	10	6.7	114	76.5	35	23.5
Elbows	121	81.2	28	18.8	146	98.0	3	2.0	137	91.9	12	8.1
Wrists/hands	77	51.7	72	48.3	135	90.6	14	9.4	108	72.5	41	27.5
Upper back	79	53.0	70	47.0	136	91.3	13	8.7	111	74.5	38	25.5
Lower back	51	34.2	98	65.8	125	83.9	24	16.1	86	57.7	63	42.3
Hips or thighs	97	65.1	52	34.9	143	96.0	6	4.0	126	84.6	23	15.4
Knees	99	66.4	50	33.6	138	92.6	11	7.4	126	84.6	23	15.4
Ankles/feet	87	58.4	62	41.6	135	90.6	14	9.4	107	71.8	42	28.2

Regarding the intensity of MSP in the last seven days, a minimum score of 4 (moderate pain) and a maximum of 10 (severe/unbearable pain) were found according to the anatomical location. The hip/thigh region showed the highest MSP intensity (7.64 ± 1.76), followed by neck (7.29 ± 2.05), ankles and feet (7.28 ± 2.230), elbows (7.25 ± 2.137), lower back (7.22 ± 1.85), shoulders (7.21 ± 1.90), wrists and hands (7.10 ± 2.08), upper back (7.03 ± 2.14), and knees (6.35 ± 2.38).

Tables 2, 3, and 4 present the sociodemographic, occupational, and health factors associated with MSP in the last seven days according to the body segment. The data presented in the tables correspond to the reports of MSP symptoms, which, added to the answers of those not reporting MSP symptoms, totals the census sample of 149 cleaning workers.

**Table 2:** Prevalence of musculoskeletal pain in the last seven days among cleaning workers according to anatomical region and sociodemographic variables, Santa Maria, Rio Grande do Sul, Brazil, 2019 (N=149)

Sociodemographic variables	Neck		Shoulders		Elbows		Wrists/hands		Upper back		Lower back		Hip/thighs		Knees		Ankles/feet	
	Yes		Yes		Yes		Yes		Yes		Yes		Yes		Yes		Yes	
	n	%	n	%	n	%	n	%	n	%	n	%	n	%	n	%	n	%
Sex																		
Women	26	19.4	33	24.6	25	18.7	37	27.6	64	47.8	57	42.5	45	33.6	45	33.6	38	28.4
Men	2	13.3	2	13.3	3	20.0	4	26.7	6	40.0	6	40.0	7	46.7	5	33.3	4	26.7
*p*	0.738^**^		0.522^**^		1.000^**^		1.000^**^		0.568^*^		0.850^*^		0.313^*^		0.985^*^		1.000^**^	
Age group																		
23 to 39	9	19.6	13	28.3	5	10.9	15	32.6	19	41.3	20	43.5	18	39.1	13	28.3	11	23.9
40 to 48	12	24.0	12	24.0	10	20.0	14	28.0	29	58.0	20	40.0	18	36.0	1	38.0	20	40.0
49 to 62	7	13.5	9	17.3	13	25.0	12	23.1	22	42.3	22	42.3	16	30.8	18	34.6	11	21.2
*p*	0.394^*^		0.427^*^		0.198^*^		0.574^*^		0.176^*^		0.940^*^		0.679^*^		0.594^*^		0.078^*^	
Education																		
Primary/Middle School (complete and incomplete)	14	21.9	18	28.1	6	9.4	15	23.4	19	29.7	28	43.8	9	14.1	7	10.9	16	25.0
High School (complete and incomplete)	14	16.7	17	20.2	6	7.1	26	31.0	19	22.6	35	41.7	14	16.7	16	19.0	26	31.0
*p*	0.423^*^		0.263^*^		0622^*^		0.312^*^		0.329^*^		0.800^*^		0.665		0.177^*^		0.426	
Marital Status																		
Married or stable relationship	17	20.7	23	28.0	5	6.1	25	30.5	24	29.3	36	43.9	13	15.9	17	20.7	24	29.3
Single or no partner	11	16.4	12	17.9	7	10.4	16	23.9	14	20.9	27	40.3	10	14.9	6	9.0	18	26.9
*p*	0.503^*^		0.146^*^		0.332^*^		0.369^*^		0.243^*^		0.658^*^		0.876^*^		0.048^*^		0.746^*^	
Number of children																		
None	0	0	1	14.3	0	0	1	14.3	0	0	2	28.6	0	0	1	14.3	1	14.3
From 1 to 2	16	20	19	23.8	5	6.3	22	27.5	23	28.8	40	50.0	15	18.8	14	17.5	25	31.3
3 or more	12	20	14	23.3	6	10	17	28.3	13	21.7	20	33.3	8	13.3	7	11.7	16	26.7
*p*	0.582^**^		1.000^**^		0.730^**^		0.859^**^		0.217^**^		0.108^**^		0.504^*^		0.674^*^		0.674^*^	

* Chi-square test;

*

* Fisher’s Exact test;

*** Corrected Chi-square.

**Table 3:** Prevalence of musculoskeletal pain in the last seven days among cleaning workers according to the anatomical region and occupational variables, Santa Maria, Rio Grande do Sul, Brazil, 2019 (N=149)

Occupational variables	Neck		Shoulders		Elbows		Wrists/hands		Upper back		Lower back		Hip/thighs		Knees		Ankles/feet	
	Yes		Yes		Yes		Yes		Yes		Yes		Yes		Yes		Yes	
	n	%	n	%	n	%	n	%	n	%	n	%	n	%	n	%	n	%
Work shift																		
Daytime	20	17.5	27	23.7	9	7.9	31	27.2	30	26.3	47	41.2	18	15.8	17	14.9	33	28.9
Nighttime	8	22.9	8	22.9	3	8.6	10	28.6	8	22.9	16	45.7	5	14.3	6	17.1	9	25.7
*p*	0.482^*^		0.920^*^		1.000^**^		0.873^*^		0.681^*^		0.638^*^		0.829^*^		0.749^*^		0.710^*^	
Time in position																		
< 1 year	12	13.5	21	23.6	7	7.9	20	22.5	21	23.6	33	37.1	13	14.6	11	12.4	20	22.5
1 ≥ 2 years	4	28.6	3	21.4	2	14.3	4	28.6	4	28.6	8	57.1	4	28.6	6	42.9	7	50.0
< 2 years and < 5 years	9	33.3	6	22.2	2	7.4	11	40.7	7	25.9	13	48.1	3	11.1	4	14.8	11	40.7
>5 years	3	15.8	5	26.3	1	5.3	6	31.6	6	31.6	9	47.4	3	15.8	2	10.5	4	21.1
*p*	0.162^***^		0.913^***^		0.776^***^		0.118^***^		0.492^***^		0.418^***^		0.953^***^		0.887^***^		0.063^*^	
Daily workload																		
6 hours	8	11.9	15	22.4	7	10.4	17	25.4	16	23.9	24	35.8	10	14.9	9	13.4	15	22.4
8 hours	7	20.0	8	22.9	2	5.7	10	28.6	9	25.7	18	51.4	7	20.0	4	11.4	13	37.1
12 hours	13	27.7	12	25.5	3	6.4	12	29.8	13	27.7	21	44.7	6	12.8	10	21.3	14	29.8
*p*	0.105^*^		0.922^*^		0.407^***^		0.863^*^		0.901^*^		0.293^*^		0.661^*^		0.394^*^		0.278^*^	
Leisure time																		
No	10	19.6	15	29.4	7	13.7	16	31.4	17	33.3	23	45.1	11	21.6	7	13.7	18	35.3
Yes	13	21.3	15	24.6	2	3.3	12	19.7	14	23.0	23	37.7	6	9.8	7	115	13	21.3
Sometimes	5	13.5	5	13.5	3	8.1	13	35.1	7	18.9	17	45.9	6	16.2	9	24.3	11	29.7
*p*	0.621^***^		0.214^***^		0.258^***^		0.188^*^		0.259^*^		0.640^*^		0.229^*^		0.214^*^		0.254^*^	
Number of people in the work schedule																		
Sufficient	9	11.4	14	17.7	5	6.3	17	21.5	19	24.1	33	41.8	12	15.2	9	11.4	20	25.3
Insufficient	18	26.5	20	29.4	6	8.8	22	32.4	18	26.5	29	42.6	10	14.7	13	19.1	20	29.4
*p*	0.019^*^		0.094^*^		0.567^*^		0.138^*^		0.736		0.915^*^		0.935^*^		0.191^*^		0.578^*^	
Second job																		
No	19	17.4	27	24.8	7	6.4	28	25.7	31	28.4	47	43.1	18	16.5	14	12.8	29	26.6
Yes	9	22.5	8	20.0	5	12.5	13	32.5	7	17.5	16	40.0	5	12.5	9	22.5	13	32.5
*p*	0.483^*^		0.543^*^		0.306^**^		0.409^*^		0.175^*^		0.733^*^		0.548^*^		0.148^*^		0.479^*^	
Training on ergonomic risks																		
No	15	22.4	18	26.9	8	11.9	17	25.4	18	26.9	31	46.3	8	11.9	8	11.9	16	23.9
Yes	13	15.9	17	20.7	4	4.9	24	29.3	20	24.4	32	39.0	15	18.3	15	18.3	26	31.7
*p*	0.310^*^		0.380^*^		0.115^*^		0.596^*^		0.730^*^		0.373^*^		0.286^*^		0.286		0.291^*^	

* Chi-square test;

*

* Fisher’s Exact test;

*** Corrected Chi-square.

**Table 4:** Prevalence of musculoskeletal pain in the last seven days among cleaning workers, according to anatomical region and health variables, Santa Maria, Rio Grande do Sul, Brazil, 2019 (N=149)

Health variables	Neck		Shoulders		Elbows		Wrists/hands		Upper back		Lower back		Hip/thighs		Knees		Ankles/feet	
	Yes		Yes		Yes		Yes		Yes		Yes		Yes		Yes		Yes	
	n	%	n	%	n	%	n	%	n	%	n	%	n	%	n	%	n	%
Smoking																		
No, never smoked	19	20.4	22	23.7	6	6.5	27	29.0	26	28.0	40	43.0	15	16.1	14	15.1	24	25.8
Yes. I smoke.	4	12.9	6	19.4	3	9.7	3	9.7	6	19.4	13	41.9	4	12.9	4	12.9	12	38.7
I quit smoking.	5	20.0	7	28.0	3	12	11	44.0	6	24.0	10	40.0	4	16.0	5	20.0	6	24.0
*p*	0.738^***^		0.749^***^		0.331^*^		0.015^*^		0.625^*^		0.963^*^		0.882^***^		0.658^***^		0.337^***^	
Alcohol consumption																		
No	15	14.6	22	21.4	7	6.8	24	23.3	23	22.3	40	38.8	13	12.6	10	9.7	26	25.2
Sometimes	13	28.3	13	28.3	5	10.9	17	37.0	15	32.6	23	50.0	10	21.7	13	28.3	16	34.8
*p*	0.048^*^		0.359^*^		0.515^**^		0.085^*^		0.184^*^		0.202^*^		0.155^*^		0.004^*^		0.232^*^	
Use of medication																		
No	11	13.9	16	20.3	6	7.6	14	17.7	15	19.0	31	39.2	12	15.2	12	15.2	20	25.3
Yes	17	24.3	19	27.1	6	8.6	27	38.6	23	32.9	32	45.7	11	15.7	11	15.7	22	31.4
*p*	0.106^*^		0.322^*^		0.827^*^		0.004^*^		0.053^*^		0.425^*^		0.930^*^		0.930^*^		0.408^*^	
Indication for medication (n=72)																		
Medical prescription	13	20.3	14	21.9	5	7.8	22	34.4	19	29.7	26	40.6	8	12.5	10	15.6	20	31.3
Self-medication	4	50.0	5	62.5	1	12.5	5	62.5	4	50.0	7	87.5	3	37.5	1	12.5	2	25.0
*p*	0.083^**^		0.026^**^		0.520^**^		0.142^*^		0.257^**^		0.020^**^		0.098^**^		1.000^**^		1.000^**^	
Medical diagnosis of at least one disease																		
No	13	14.6	22	24.7	6	6.7	21	23.6	22	24.7	36	40.4	13	14.6	13	14.6	23	25.8
Yes	15	25.0	13	21.7	6	10	20	33.3	16	26.7	27	45.0	10	16.7	10	16.7	19	31.7
*p*	0.111^*^		0.666^*^		0.546^**^		0.192^*^		0.789^*^		0.581^*^		0.733^*^		0.733^*^		0.43^**^	
Daily hours of sleep (n=146)																		
Less than 8 hours	20	20.0	23	23.0	8	8.0	33	33.0	30	30.0	44	44.0	18	18.0	16	16.0	34	34.0
8 or more hours	8	17.4	11	23.9	4	8.7		15.2	8	17.4	18	39.1	5	10.9	7	15.2	8	17.4
*p*	0.710		0.903		0.887		0.025		0.107		0.580		0.272		0.904		0.039	
Physical exercise																		
No	19	19.8	23	24.0	11	11.5	28	29.2	28	29.2	45	46.9	17	17.7	18	18.8	33	34.4
Yes	9	21.4	12	28.6	0	0.0	12	28.6	7	16.7	14	33.3	4	9.5	4	9.5	7	16.7
Sometimes	0	0	0	0	1	9.1	1	9.1	3	27.3	4	36.4	2	18.2	1	9.1	2	18.2
*p*	0.313^***^		0.351^***^		0.043^*^		0.292^***^		0.321^***^		0.182^***^		0.498^***^		0.162^***^		0.041^***^	
Body Mass Index^*^																		
Up to 24.9 (underweight up to healthy weight)	5	13.5	8	21.6	2	5.4	5	13.5	10	27.0	16	43.2	7	18.9	4	10.8	9	24.3
25 to 29.9 (overweight)	13	22.4	14	24.1	4	6.9	19	32.8	14	24.1	26	44.8	7	12.1	9	15.5	16	27.6
30 to 40 (obesity)	10	18.5	13	24.1	6	11.1	17	31.5	14	25.9	21	38.9	9	16.7	10	18.5	17	31.5
*p*	0.555^*^		0.953^*^		0.307^***^		0.088^*^		0.948^*^		0.809		0.634^*^		0.607^*^		0.751^*^	
Waist-hip ratio																		
Normal	9	26.5	8	23.5	2	5.9	8	23.5	7	20.6	17	50.0	7	20.6	6	17.6	8	23.5
High	19	16.5	27	23.5	10	8.7	33	28.7	31	27	46	40.0	16	13.9	17	14.8	34	29.6
*p*	0.192^*^		0.995		0.735^**^		0.553		0.454		0.300^*^		0.344^*^		0.685		0.492^*^	

* Chi-square test;

*

* Fisher’s Exact test;

*** Corrected Chi-square.

A statistically significant association was found between MSP in the knee region in the last seven days and married workers or in a stable relationship (p=0.048) (Table 2).

MSP in the neck region in the last seven days was statistically associated with dissatisfaction with the number of people on the work schedule (p=0.019) (Table 3).

MSP in the neck in the last seven days was associated with the occasional consumption of alcohol (p=0.048); pain in the shoulders or lower back was associated with self-medication (p=0.026 and p=0.020, respectively); pain in the elbows was associated with a lack of physical exercise (sedentary lifestyle) (p=0.043); pain in the wrists/hands was associated with being a former smoker (I have smoked, but quit) (p=0.015) and taking medications (p=0.004). Pain in the lower limbs, such as in the knees, was associated with the occasional consumption of alcohol (p=0.004); and pain in the ankles/feet was associated with a sedentary lifestyle (p=0.041) and sleeping less than eight hours a day (p=0.039).

## DISCUSSION

MSP among hospital cleaning workers in the last seven days was prevalent in the lumbar region (42.3%), followed by ankles/feet (28.2%), wrists/hands (27.5%), upper back (25.5%), and shoulders (23.5%). Such conditions are of concern, as they reflect pain experienced recently and possibly associated with the cleaning process. A study addressing outsourced cleaning workers from a public university found that 22.7% had already been on sick leave due to musculoskeletal diseases. Furthermore, 81.8% reported MSP related to the type of activity and working conditions^(15)^.

According to the *Pesquisa Nacional de Saúde* [National Health Survey]^(16)^, approximately 34.3 million Brazilians (21.6%) reported chronic back pain; 2.5% of the population presents a diagnosed work-related musculoskeletal disorder. These conditions may be related to maintaining standing postures for prolonged periods and overloading the spine. Furthermore, the manual lifting of loads, bending, twisting, and tilting of the spine associated with repetitive movements and poor posture may result in painful symptoms^(17)^.

In this study, self-medication was associated with low back pain (p=0.020) and MSP in the shoulders (p=0.026). Hence, the hypothesis concerning the relationship between self-medication and pain is supported here, as the cleaning workers reported using analgesic and anti-inflammatory medications to alleviate pain.

MSP in ankles and feet, the second most affected region, indicates prolonged orthostatism as an important factor to be considered. Remaining in a standing position without pause is often associated with footwear and rigid surfaces, which cause increased pressure on the plantar muscles and a low threshold for vascular symptoms, which may result in pain^(18)^. Thus, standing for long periods might represent a biomechanical risk to cleaning workers, exposing body-supporting muscle and joint structures^(18)^. A strategy to minimize MSP in ankles and feet is to alternate activities that require prolonged orthostatism, such as the hygiene and cleaning of environments, with sitting or walking, to alternate between different muscle groups^(19)^.

The association between MSP in ankles and feet and a sedentary lifestyle (p=0.041) results from workers not performing regular physical activity, as it results in insufficient physical fitness to perform highly demanding tasks. In this sense, physical inactivity may lead to muscle atrophy as well as atrophy of supporting connective tissues. Additionally, a sedentary lifestyle is associated with increasingly stiff tendons, fascia, ligaments, and muscles^(20)^. Research indicates that guided physical activity is effective in maintaining and restoring work capacity, increasing oxygenation at the cellular level, reducing stress, and improving self-esteem^(20,21)^.

MSP in wrists/hands, the third most frequently affected body segment, occurs because cleaning workers’ tasks require the constant use of the upper limbs, with activities involving the use of hands to clean and disinfect surfaces, countertops, and equipment^(21)^. Moreover, the quality and type of equipment (squeegee extender, mop, and washing machine) determines the effort required from wrists and hands.

Factors that influence the occurrence of hand injuries include the weight and type of load^(21)^. When the shape of a load/equipment is similar to the hands’ anatomy, there is greater contact with the object, which allows for a firmer grip. In this case, less force is needed to facilitate the process. At the same time, a larger object requires more force to be exerted, and a greater number of body segments are recruited to stabilize the movement^(22)^.

Smokers and former smokers presented a higher frequency of MSP in the lumbar spine (81.9%), ankles/feet (62.7%), and hips/thighs (61.8%). Such an association may be related to the cigarette components changing the pH and nutrition of the intervertebral discs, predisposing them to herniations. Additionally, there is a connection between tobacco use and decreased resistance of the stabilizing muscles of the lumbar spine, predisposing to pain. Nicotine possibly affects the central nervous system and causes vasoconstriction and decreased cellular oxygenation, interfering with one’s perception of pain^(23)^.

The fourth most frequently affected area was the upper back/thoracic spine (25.5%). This is the second region of the spine, positioned between the cervical and lumbar spines. Cleaning work requires flexing and rotating the spine, which, when associated with lifting weights (garbage bags), is a significant aggravating factor of MSP in the thoracic spine. Similar to urban street sweeping work, symptoms in this region reduce work capacity^(24)^.

Another important risk factor is performing rapid repetitive movements, such as cleaning under hospital beds. Authors^(23,24)^ report that uncomfortable, restricted, asymmetrical, repeated, and prolonged work postures, as well as extreme and repetitive movements and the use of excessive force, may lead to tissue overload, exceeding stress limits, causing tissue injuries due to overloading of the musculoskeletal structures, especially the spine.

Regarding MSP in the shoulder region, cleaning workers remain for long periods with their arms raised without support. This finding is also explained by the high rate of repetitive movements and gestural biomechanics, which are characteristic of sweeping, handling loads, raising the upper limbs, and placing garbage in the cart^(24)^.

Even though, 38.9% of the cleaning workers were overweight (BMI of 25 to 29.9 kg/m^2^), and 36.2% presented obesity classified in classes I, II, and III, no significant association was found between obesity and MSP in the body segments. Thus, it is important to consider such conditions as comorbidities and a significant risk factor for MSP. Despite the workers performing activity-induced energy expenditure, reaching increased heart rate, and reporting time for leisure, these are insufficient to prevent overweight. Therefore, regular physical activity must be encouraged to improve strength, flexibility, and localized muscular resistance, promoting quality of life and increasing work capacity^(25)^.

This study’s results suggest that exposure to ergonomic factors may cause tissue overload and exceed its stress limits, causing tissue injuries due to inadequate strain and overload on musculoskeletal structures, especially the spine. Consequently, MSP occurs, and a more pronounced impact is verified when two or more of these risk factors are combined in a single task.

### Study limitations

One limitation of this study is that it is impossible to determine a cause-and-effect relationship between exposure and outcome, a condition related to cross-sectional investigations.

### Contributions to the nursing and health fields

In addition to encouraging reflections and a discussion on the factors associated with exposure to MSP in cleaning jobs, the scientific knowledge obtained here concerns the health of support workers, such as hospital cleaning staff. These results are expected to facilitate understanding of the ergonomic risk factors imposed on cleaning workers and which strategies can minimize the occurrence of MSP, and most of all, facilitate the process of transforming and promoting healthy work environments.

## CONCLUSIONS

The hospital cleaning workers addressed here performed their work tasks, experiencing considerable musculoskeletal symptoms, mainly low back pain. The factors associated with MSP are related to occupational variables, health, and lifestyle, such as: dissatisfaction with the number of people on the work schedule; the occasional consumption of alcohol, and neck pain; self-medication and pain in the lower back and shoulders; sedentary lifestyle and elbow pain; being a former smoker, using medication, and pain in wrists/hands; the occasional consumption of alcohol and knee pain; and sedentary lifestyle, the amount of sleep hours, and pain in ankles/feet.

Managers of facilities where cleaning workers operate are recommended to implement actions to reduce ergonomic risks and negative work outcomes, acknowledge these workers, and invest in training and programs to improve the work process, to minimize the exposure of these individuals to ergonomic risks and MSP. Additionally, the working conditions of hospital cleaning workers must be improved to decrease their exposure to MSP and promote healthy work conditions. Health education programs and recommendations to improve work processes, aimed at cleaning workers and their management, might minimize exposure to MSP. Finally, we suggest developing studies with longitudinal and experimental designs, including larger samples and other hospital settings.
