# Supplementary material for: Prevalence and factors associated with musculoskeletal pain among hospital cleaning staff
Source: Rev Bras Enferm. 2024 Dec 16;77(6):e20230237. doi: 10.1590/0034-7167-2023-0237 (PMC11654551; doi:10.1590/0034-7167-2023-0237)
Supplement: Translation

# Translation: Prevalência e fatores associados à dor musculoesquelética em trabalhadores do serviço hospitalar de limpeza

**DOI:** 10.1590/0034-7167-2023-0237pt

**Authors:** Emanuelli Mancio Ferreira da Luz, Oclaris Lopes Munhoz, Patrícia Bitencourt Toscani Greco, Bruna Xavier Morais, Silviamar Camponogara, Tânia Solange Bosi de Souza Magnago

**Affiliations:** I Universidade Federal do Rio Grande. Rio Grande, Rio Grande do Sul, Brasil; II Universidade Federal de Santa Maria. Santa Maria, Rio Grande do Sul, Brasil

**Keywords:** Saúde do Trabalhador, Dor Musculoesquelética, Transtornos Traumáticos Cumulativos, Serviço Hospitalar de Limpeza, Serviços Hospitalares.

## Abstract

**Objetivos:**

verificar a prevalência e os fatores associados à dor musculoesquelética em trabalhadores do serviço hospitalar de limpeza.

**Métodos:**

estudo transversal, realizado com trabalhadores de limpeza de um hospital de ensino do Sul do Brasil. Utilizaram-se questionário com variáveis sociodemográficas, laborais e de saúde, o *Nordic Musculoskeletal Questionnaire* e o Diagrama de Corlett e Manenica. Realizou-se análise bivariada.

**Resultados:**

participaram 149 trabalhadores. Prevaleceu dor musculoesquelética na coluna lombar no último ano (65,8%) e últimos sete dias (42,3%). Constataram-se associações entre automedicação e dor na parte inferior das costas (p=0,020) e ombros (p=0,026); sedentarismo, oito horas de sono diárias e dor nos tornozelos (p=0,041) e pés (p=0,039); ex-tabagismo, uso de medicamento e dor nos punhos (p=0,015) e mãos (p=0,004).

**Conclusões:**

prevaleceram lombalgias associadas a hábitos de saúde e vida. Um programa de educação em saúde e recomendações de melhorias nos processos de trabalho podem minimizar a exposição à dor musculoesquelética.

## INTRODUÇÃO

A dor musculoesquelética (DME) ocorre devido ao uso excessivo do sistema musculoesquelético associado à falta de tempo para recuperação do mesmo. Pode ser expressa por meio do autorrelato de sintomas como dor (em ossos, articulações, músculos ou estruturas circunjacentes), fadiga e parestesia^(1)^. Trata-se de um importante agravo físico à saúde, e corresponde a um dos principais acometimentos em trabalhadores dos mais diversos grupos ocupacionais, os quais estão expostos à alta demanda física e a movimentos constantes^(2)^.

No contexto do serviço hospitalar de limpeza (SHL), os trabalhadores possuem uma demanda laboral dinâmica e intensa^(3)^, podendo ocasionar a sobrecarga do sistema musculoesquelético e o consequente desgaste físico, resultando em DME^(4)^. Nessa perspectiva, a exposição dos trabalhadores de limpeza hospitalar à DME pode estar associada ao seu processo de trabalho. Este é caracterizado pela realização de atividades manuais e repetitivas, com pouco auxílio mecânico, uso de força e ritmo intenso de trabalho^(5)^. Ainda, as atividades de limpeza são caracterizadas por posturas corporais não ergonômicas associadas a tarefas com frequente manuseio de carga e trabalho muscular estático nas atividades de puxar, empurrar, ficar em pé e andar^(3)^.

Estas exposições, apesar de reconhecidamente evitáveis, são responsáveis por ocasionar sobrecarga do sistema musculoesquelético e sintomatologia da DME. Logo, constituem a causa primordial de diferentes graus de limitação e incapacidade para o trabalho, queda na qualidade dos serviços, correspondendo ao maior percentual de absenteísmo laboral que, por vezes, é mais prolongado e recorrente, quando comparado a outros agravos^(6)^.

Nesta perspectiva, de acordo com os registros da Previdência Social do Brasil, na última década, os grupos diagnósticos com maiores prevalências de benefícios do tipo auxílio-doença foram doenças osteomusculares^(7)^. Desse modo, o benefício auxílio-doença, por doenças do sistema osteomuscular e do tecido conjuntivo, em 2020, foi concedido a 150.361 e, em 2021, a 303.399 trabalhadores urbanos e rurais^(7)^. Em trabalhadores do SHL, encontra-se uma prevalência de 70,1%, sendo 25,5% os casos de sintomatologia de intensidade forte e insuportável^(8)^.

Para tanto, pesquisas com vistas a oferecer subsídios sobre a exposição e o acometimento musculoesquelético do trabalhador de limpeza, especificamente atuante em ambiente hospitalar, são importantes, porém escassas. Somado a isso, tem se mostrado uma tendência a atuação do enfermeiro na governança em higiene e limpeza, e nas organizações hospitalares, com vistas a garantir o dimensionamento suficiente para a segurança dos trabalhadores e/ou clientes, bem como a limpeza dos setores, com consequente minimização das infecções e acidentes de trabalho^(9)^. Outrossim, merece destaque a interface entre SHL e enfermagem, devido à contemporaneidade da temática e ao desafio para o enfermeiro^(9)^, visto que investir em cuidados à saúde desses trabalhadores e contribuir para a sua visibilidade nas instituições torna-se espaço de inovação em sua atuação.

## OBJETIVOS

Verificar a prevalência e os fatores associados à DME em trabalhadores do SHL.

## MÉTODOS

### Aspectos éticos

Estudo aprovado pelo Comitê de Ética em Pesquisa da instituição proponente em 14 de agosto de 2018. Os trabalhadores receberam orientações referentes aos objetivos da pesquisa, e, após aceite, assinaram duas vias do Termo de Consentimento Livre e Esclarecido (TCLE). Foram respeitados os aspectos éticos, conforme Resolução n^o^ 466/2012 do Conselho Nacional de Saúde, garantindo a voluntariedade da participação, o anonimato dos participantes e o sigilo dos dados obtidos.

### Tipo de estudo

Estudo transversal, que seguiu as recomendações do *STrengthening the Reporting of OBservational Studies in Epidemiology* (STROBE)^(10)^ para clareza e transparência da redação.

### Local de estudo

Pesquisa desenvolvida em hospital público de ensino do Sul do Brasil, local onde estão alocados os trabalhadores do SHL, contratados por uma empresa de prestação de serviços terceirizados. No período do estudo, 152 trabalhadores do SHL realizavam atividades de limpeza da área física e de 403 leitos de internação.

### Critérios de seleção e definição da amostra

Foram incluídos os trabalhadores do SHL com tempo de trabalho mínimo de 30 dias. Esta delimitação baseou-se no pressuposto de que este seria o tempo mínimo para que o trabalhador do SHL estivesse adaptado à empresa e ao setor e, com a realização das atividades diárias, pudesse observar a presença de DME em seu contexto laboral. Foram excluídos os trabalhadores em licença de saúde ou afastamento, por qualquer motivo, durante a coleta de dados. Assim, constituiu-se uma amostra censitária de 149 (98%) participantes que responderam ao questionário. As perdas (n=3; 2%) resultaram de não aceitação em participar do estudo (n=2) e ausência na coleta de dados (n=1).

### Coleta de dados

A coleta de dados ocorreu em julho de 2019, nos turnos de trabalho dos investigados, em local reservado, de maneira que pudessem se ausentar de suas atividades laborais sem ônus, com a devida autorização dos gestores do SHL. Foi realizada por estudantes da pós-graduação e bolsistas de iniciação científica previamente capacitados pela autora (teste piloto da aplicação do questionário e do protocolo de coleta de dados). Após a capacitação, o convite foi realizado aos trabalhadores de forma individual, com base na listagem de colaboradores e nas escalas de trabalho fornecidas pela empresa. Em caso de ausência de algum trabalhador no momento da coleta de dados, realizou-se contato via telefonema para o setor, com agendamento de uma nova data.

Utilizaram-se um questionário autoaplicável, contendo a caracterização sociodemográfica (sexo, faixa etária, escolaridade, situação conjugal, número de filhos), laboral (turno, tempo de trabalho na função, carga horária diária, tempo para o lazer, satisfação com o número de pessoas na escala, outro emprego e realização de treinamento sobre riscos ergonômicos), de saúde (tabagismo, consumo de bebida alcoólica, uso de medicação, diagnóstico médico de doença, horas de sono diárias, prática de atividade física e medidas antropométricas como peso, altura, circunferência abdominal e quadril, e Índice de Massa Corporal (IMC)), bem como a versão brasileira do *Nordic Musculoskeletal Questionnaire* (NMQ)^(11)^, para a avaliação do desfecho da DME, e o Diagrama de Corlett e Manenica^(12)^. Este mapa de regiões corporais foi utilizado como uma ferramenta auxiliar, fornecida ao investigado para a demarcação dos segmentos e da intensidade de sua dor e/ou desconforto corporal^(12)^. Ambos instrumentos são de acesso livre^(11,12)^.

Definiram-se como portadores de DME aqueles trabalhadores que responderam afirmativamente à pergunta do NMQ^(11)^: no último ano e nos últimos sete dias, você teve alguma dor ou desconforto em (pescoço, ombros, cotovelos, pulso ou mão, coluna torácica, coluna lombar, coxas, pernas, joelhos e tornozelos)? Além disso, o trabalhador pôde relatar a ocorrência de impedimento/afastamento das funções laborais no último ano no NMQ^(11)^ e indicar a área da dor e/ou desconforto osteomuscular nas 25 regiões da figura de um corpo humano, dividido em regiões direita e esquerda, bem como a intensidade de sua ocorrência^(12)^.

### Análise e tratamento de dados

Os dados foram inseridos no *software* Epi Info^®^, versão 6.4, com dupla digitação independente. Após a verificação de erros e inconsistências, os dados foram analisados no programa *Statistical Package for the Social Sciences* (SPSS^®^, SPSS Inc, Chicago), versão 18.0, utilizando-se estatística descritiva e estatística inferencial. As variáveis categóricas foram apresentadas por meio de frequência absoluta (n) e frequência relativa (%). Já as variáveis quantitativas, de acordo com a normalidade ou não dos dados (teste Kolmogorov-Smirnov), foram descritas por medidas de posição e dispersão (média ou mediana, desvio padrão, ou intervalo interquartílico, respectivamente).

A caracterização do perfil sociodemográfico, laboral e de saúde dos trabalhadores do SHL foi realizada por meio da estatística descritiva. A DME foi analisada de forma dicotômica (presente ou ausente) em cada região anatômica, apresentando-se a frequência absoluta (N) e a frequência relativa (%). Para este estudo, devido ao tempo médio de 11 meses de trabalho na função (59,7%), para a análise bivariada, a DME foi considerada como aquela relatada nos sete dias anteriores à entrevista (variável dependente). A prevalência (P)^(13)^ de DME foi verificada de acordo com a expressão:


$$P=\frac{\text{Nº de relatos de DME em um dado local e período}}{\text{população do mesmo local e período}}\times 100$$

A intensidade da DME nos últimos sete dias, conforme a localização anatômica do Diagrama de Corlett e Manenica, foi avaliada de 0 (sem dor) a 10 (dor intensa/insuportável)^(12)^. Realizaram-se análises bivariadas para fins de identificação de associação entre DME nos últimos sete dias e as variáveis independentes (sociodemográficas, laborais, hábitos e saúde). O teste qui-quadrado de Pearson e o teste exato de Fisher foram utilizados considerando Intervalo de Confiança de 95% (p<0,05). Para a análise do IMC, foram utilizadas a fórmula peso/(altura)^2^ e a categorização, conforme os padrões internacionais de avaliação de adultos^(14)^.

## RESULTADOS

Dos 149 trabalhadores do SHL (98%) que participaram do estudo, predominaram mulheres (89,9%; n=134), na faixa etária de 49 a 62 anos (34,9%, n=52), ensino médio completo (36,2%; n=54), casados e/ou com companheiros (55%; n=82) e com um a dois filhos (53,7%). A caracterização laboral apontou prevalência de trabalhadores atuantes no diurno (76,5%; n=114), com tempo de trabalho inferior a um ano (59,7%; n=89), carga horária de seis horas diárias (45%; n=67), além de número de pessoas na escala de trabalho suficiente (53%; n=79), trabalhadores que não possuem outro vínculo empregatício (73,2%; n=109) e que não realizaram treinamentos sobre riscos ergonômicos (45%; n=67).

Em relação aos hábitos e saúde, maior percentual relatou possuir tempo para lazer (40,9%; N=61), não fazer uso de tabaco (62,4%; n=93) e de bebida alcoólica (69,1%; n=103) e utilizar algum tipo de medicamento (47%; n=70). Dentre estes, o grupo dos anti-inflamatórios (48,3%; n=34) foi o mais frequente. Com relação a possuir doença diagnosticada, 40,3% (n=60) possuem algum diagnóstico médico. Destes, os distúrbios musculoesqueléticos (tendinopatia, síndrome de túnel do carpo, epicondilite, bursite, neuropatia periférica, fissura lombar e distensão cervical) (31,2%; n=46) foram prevalentes. Ainda se evidenciaram trabalhadores do SHL com média de 6,61 horas de sono (±10,79) e sedentários (64,4%; n=96). Em relação ao IMC, 38,9% (n=58) apresentam-se com excesso de peso corporal (IMC de 25 a 29,9 kg/m^2^), sendo 36,2% (n=54) com obesidade graus I, II e III. A relação cintura/quadril apresentou uma média de 0,90 cm (±0,06), mínimo de 0,63 cm e máximo de 1,10 cm.

A prevalência de DME relatada pelos trabalhadores do SHL no último ano e nos sete dias anteriores à entrevista, segundo a localização anatômica, está apresentada na Tabela 1.

**Tabela 1:** Distribuição dos trabalhadores do serviço hospitalar de limpeza segundo a localização anatômica de dor musculoesquelética relatada nos últimos 12 meses e sete dias (*Nordic Musculoskeletal Questionnaire*), Santa Maria, Rio Grande do Sul, Brasil, 2019 (N=149)

Localização anatômica	Nos últimos 12 meses, você teve (dor, formigamento, dormência) em:				Nos últimos 12 meses, você foi impedido(a) de realizar atividades normais por causa desses problemas em:				Nos últimos sete dias, você teve algum problema em:			
	Não		Sim		Não		Sim		Não		Sim	
	n	%	n	%	n	%	n	%	n	%	n	%
Pescoço	94	63,1	55	36,9	135	90,6	14	9,4	121	81,2	28	18,8
Ombros	72	48,3	77	51,7	139	93,3	10	6,7	114	76,5	35	23,5
Cotovelos	121	81,2	28	18,8	146	98,0	3	2,0	137	91,9	12	8,1
Punhos/mãos	77	51,7	72	48,3	135	90,6	14	9,4	108	72,5	41	27,5
Parte superior das costas	79	53,0	70	47,0	136	91,3	13	8,7	111	74,5	38	25,5
Parte inferior das costas	51	34,2	98	65,8	125	83,9	24	16,1	86	57,7	63	42,3
Quadril ou xoxas	97	65,1	52	34,9	143	96,0	6	4,0	126	84,6	23	15,4
Joelhos	99	66,4	50	33,6	138	92,6	11	7,4	126	84,6	23	15,4
Tornozelos/pés	87	58,4	62	41,6	135	90,6	14	9,4	107	71,8	42	28,2

Quanto ao grau de intensidade da DME, nos últimos sete dias, conforme a localização anatômica, evidenciou-se o mínimo de 4 (dor moderada) e máximo de 10 (dor intensa/insuportável). A região do quadril/coxas obteve maior intensidade de DME (7,64 ± 1,76), seguida pelo pescoço (7,29 ± 2,05), tornozelos e pés (7,28 ± 2,230), cotovelos (7,25 ± 2,137), parte inferior das costas (7,22 ± 1,85), ombros (7,21 ± 1,90), punhos/mãos (7,10 ± 2,08), parte superior das costas (7,03 ± 2,14) e joelhos (6,35 ± 2,38).

Nas Tabelas 2, 3 e 4, apresentam-se os fatores sociodemográficos, laborais e de saúde associados à ocorrência de DME por segmento corporal nos últimos sete dias. Nestas, os dados apresentados correspondem aos relatos afirmativos de sintomatologia de DME e, que ao ser somado com os trabalhadores que não autorrelataram DME, alcançam a amostra censitária dos 149 trabalhadores do SHL.

**Tabela 2:** Prevalência de dor musculoesquelética por regiões anatômicas, nos sete dias anteriores à entrevista, em trabalhadores do serviço hospitalar de limpeza, segundo variáveis sociodemográficas, Santa Maria, Rio Grande do Sul, Brasil, 2019 (N=149)

Variáveis sociodemográficas	Pescoço		Ombros		Cotovelos		Punhos ou mãos		Parte superior das costas		Parte inferior das costas		Quadril e coxas		Joelhos		Tornozelos e pés	
	Sim		Sim		Sim		Sim		Sim		Sim		Sim		Sim		Sim	
	n	%	n	%	n	%	n	%	n	%	n	%	n	%	n	%	n	%
Sexo																		
Feminino	26	19,4	33	24,6	25	18,7	37	27,6	64	47,8	57	42,5	45	33,6	45	33,6	38	28,4
Masculino	2	13,3	2	13,3	3	20,0	4	26,7	6	40,0	6	40,0	7	46,7	5	33,3	4	26,7
*p*	0,738^**^		0,522^**^		1,000^**^		1,000^**^		0,568^*^		0,850^*^		0,313^*^		0,985^*^		1,000^**^	
Faixa etária																		
23 a 39 anos	9	19,6	13	28,3	5	10,9	15	32,6	19	41,3	20	43,5	18	39,1	13	28,3	11	23,9
40 a 48 anos	12	24,0	12	24,0	10	20,0	14	28,0	29	58,0	20	40,0	18	36,0	1	38,0	20	40,0
49 a 62 anos	7	13,5	9	17,3	13	25,0	12	23,1	22	42,3	22	42,3	16	30,8	18	34,6	11	21,2
*p*	0,394^*^		0,427^*^		0,198^*^		0,574^*^		0,176^*^		0,940^*^		0,679^*^		0,594^*^		0,078^*^	
Escolaridade																		
Ensino fundamental completo e incompleto	14	21,9	18	28,1	6	9,4	15	23,4	19	29,7	28	43,8	9	14,1	7	10,9	16	25,0
Ensino médio completo e incompleto	14	16,7	17	20,2	6	7,1	26	31,0	19	22,6	35	41,7	14	16,7	16	19,0	26	31,0
*p*	0,423^*^		0,263^*^		0622^*^		0,312^*^		0,329^*^		0,800^*^		0,665		0,177^*^		0,426	
Situação conjugal																		
Casado ou com companheiro	17	20,7	23	28,0	5	6,1	25	30,5	24	29,3	36	43,9	13	15,9	17	20,7	24	29,3
Solteiro ou sem companheiro	11	16,4	12	17,9	7	10,4	16	23,9	14	20,9	27	40,3	10	14,9	6	9,0	18	26,9
*p*	0,503^*^		0,146^*^		0,332^*^		0,369^*^		0,243^*^		0,658^*^		0,876^*^		0,048^*^		0,746^*^	
Número de filhos																		
Nenhum	0	0	1	14,3	0	0	1	14,3	0	0	2	28,6	0	0	1	14,3	1	14,3
De 1 a 2	16	20	19	23,8	5	6,3	22	27,5	23	28,8	40	50,0	15	18,8	14	17,5	25	31,3
De 3 ou mais	12	20	14	23,3	6	10	17	28,3	13	21,7	20	33,3	8	13,3	7	11,7	16	26,7
*p*	0,582^**^		1,000^**^		0,730^**^		0,859^**^		0,217^**^		0,108^**^		0,504^*^		0,674^*^		0,674^*^	

* Teste qui-quadrado;

*

* Teste exato de Fisher;

*** Teste qui-quadrado com correção.

**Tabela 3:** Prevalência de dor musculoesquelética por regiões anatômicas, nos sete dias anteriores à entrevista, em trabalhadores do serviço hospitalar de limpeza, segundo variáveis laborais, Santa Maria, Rio Grande do Sul, Brasil, 2019 (N=149)

Variáveis laborais	Pescoço		Ombros		Cotovelos		Punhos ou mãos		Parte superior das costas		Parte inferior das costas		Quadril e coxas		Joelhos		Tornozelos e pés	
	Sim		Sim		Sim		Sim		Sim		Sim		Sim		Sim		Sim	
	n	%	n	%	n	%	n	%	n	%	n	%	n	%	n	%	n	%
Turno de trabalho																		
Diurno	20	17,5	27	23,7	9	7,9	31	27,2	30	26,3	47	41,2	18	15,8	17	14,9	33	28,9
Noturno	8	22,9	8	22,9	3	8,6	10	28,6	8	22,9	16	45,7	5	14,3	6	17,1	9	25,7
*p*	0,482^*^		0,920^*^		1,000^**^		0,873^*^		0,681^*^		0,638^*^		0,829^*^		0,749^*^		0,710^*^	
Tempo de trabalho nesta função																		
< 1 ano	12	13,5	21	23,6	7	7,9	20	22,5	21	23,6	33	37,1	13	14,6	11	12,4	20	22,5
1 ≥ 2 anos	4	28,6	3	21,4	2	14,3	4	28,6	4	28,6	8	57,1	4	28,6	6	42,9	7	50,0
< 2 anos e < 5 anos	9	33,3	6	22,2	2	7,4	11	40,7	7	25,9	13	48,1	3	11,1	4	14,8	11	40,7
>5 anos	3	15,8	5	26,3	1	5,3	6	31,6	6	31,6	9	47,4	3	15,8	2	10,5	4	21,1
*p*	0,162^***^		0,913^***^		0,776^***^		0,118^***^		0,492^***^		0,418^***^		0,953^***^		0,887^***^		0,063^*^	
Carga horária diária																		
6 horas	8	11,9	15	22,4	7	10,4	17	25,4	16	23,9	24	35,8	10	14,9	9	13,4	15	22,4
8 horas	7	20,0	8	22,9	2	5,7	10	28,6	9	25,7	18	51,4	7	20,0	4	11,4	13	37,1
12 horas	13	27,7	12	25,5	3	6,4	12	29,8	13	27,7	21	44,7	6	12,8	10	21,3	14	29,8
*p*	0,105^*^		0,922^*^		0,407^***^		0,863^*^		0,901^*^		0,293^*^		0,661^*^		0,394^*^		0,278^*^	
Tempo para o lazer																		
Não	10	19,6	15	29,4	7	13,7	16	31,4	17	33,3	23	45,1	11	21,6	7	13,7	18	35,3
Sim	13	21,3	15	24,6	2	3,3	12	19,7	14	23,0	23	37,7	6	9,8	7	115	13	21,3
Às vezes	5	13,5	5	13,5	3	8,1	13	35,1	7	18,9	17	45,9	6	16,2	9	24,3	11	29,7
*p*	0,621^***^		0,214^***^		0,258^***^		0,188^*^		0,259^*^		0,640^*^		0,229^*^		0,214^*^		0,254^*^	
Número de pessoas na escala de trabalho																		
Suficiente	9	11,4	14	17,7	5	6,3	17	21,5	19	24,1	33	41,8	12	15,2	9	11,4	20	25,3
Insuficiente	18	26,5	20	29,4	6	8,8	22	32,4	18	26,5	29	42,6	10	14,7	13	19,1	20	29,4
*p*	0,019^*^		0,094^*^		0,567^*^		0,138^*^		0,736		0,915^*^		0,935^*^		0,191^*^		0,578^*^	
Outro emprego																		
Não	19	17,4	27	24,8	7	6,4	28	25,7	31	28,4	47	43,1	18	16,5	14	12,8	29	26,6
Sim	9	22,5	8	20,0	5	12,5	13	32,5	7	17,5	16	40,0	5	12,5	9	22,5	13	32,5
*p*	0,483^*^		0,543^*^		0,306^**^		0,409^*^		0,175^*^		0,733^*^		0,548^*^		0,148^*^		0,479^*^	
Treinamentos sobre riscos ergonômicos																		
Não	15	22,4	18	26,9	8	11,9	17	25,4	18	26,9	31	46,3	8	11,9	8	11,9	16	23,9
Sim	13	15,9	17	20,7	4	4,9	24	29,3	20	24,4	32	39,0	15	18,3	15	18,3	26	31,7
*p*	0,310^*^		0,380^*^		0,115^*^		0,596^*^		0,730^*^		0,373^*^		0,286^*^		0,286		0,291^*^	

* Teste qui-quadrado;

** Teste exato de Fisher;

*** Teste qui-quadrado com correção.

**Tabela 4:** Prevalência de dor musculoesquelética por regiões anatômicas, nos sete dias anteriores à entrevista, em trabalhadores do serviço hospitalar de limpeza, segundo variáveis de saúde, Santa Maria, Rio Grande do Sul, Brasil, 2019 (N=149)

Variáveis de saúde	Pescoço		Ombros		Cotovelos		Punhos ou mãos		Parte superior das costas		Parte inferior das costas		Quadril e coxas		Joelhos		Tornozelos e pés	
	Sim		Sim		Sim		Sim		Sim		Sim		Sim		Sim		Sim	
	n	%	n	%	n	%	n	%	n	%	n	%	n	%	n	%	n	%
Tabagismo																		
Não, nunca fumei	19	20,4	22	23,7	6	6,5	27	29,0	26	28,0	40	43,0	15	16,1	14	15,1	24	25,8
Sim, fumo	4	12,9	6	19,4	3	9,7	3	9,7	6	19,4	13	41,9	4	12,9	4	12,9	12	38,7
Fumei, mas parei	5	20,0	7	28,0	3	12	11	44,0	6	24,0	10	40,0	4	16,0	5	20,0	6	24,0
*p*	0,738^***^		0,749^***^		0,331^*^		0,015^*^		0,625^*^		0,963^*^		0,882^***^		0,658^***^		0,337^***^	
Uso de álcool																		
Não	15	14,6	22	21,4	7	6,8	24	23,3	23	22,3	40	38,8	13	12,6	10	9,7	26	25,2
Às vezes	13	28,3	13	28,3	5	10,9	17	37,0	15	32,6	23	50,0	10	21,7	13	28,3	16	34,8
*p*	0,048^*^		0,359^*^		0,515^**^		0,085^*^		0,184^*^		0,202^*^		0,155^*^		0,004^*^		0,232^*^	
Uso de medicação																		
Não	11	13,9	16	20,3	6	7,6	14	17,7	15	19,0	31	39,2	12	15,2	12	15,2	20	25,3
Sim	17	24,3	19	27,1	6	8,6	27	38,6	23	32,9	32	45,7	11	15,7	11	15,7	22	31,4
*p*	0,106^*^		0,322^*^		0,827^*^		0,004^*^		0,053^*^		0,425^*^		0,930^*^		0,930^*^		0,408^*^	
Indicação do medicamento (n=72)																		
Indicação médica	13	20,3	14	21,9	5	7,8	22	34,4	19	29,7	26	40,6	8	12,5	10	15,6	20	31,3
Automedicação	4	50,0	5	62,5	1	12,5	5	62,5	4	50,0	7	87,5	3	37,5	1	12,5	2	25,0
*p*	0,083^**^		0,026^**^		0,520^**^		0,142^*^		0,257^**^		0,020^**^		0,098^**^		1,000^**^		1,000^**^	
Diagnóstico médico de alguma doença																		
Não	13	14,6	22	24,7	6	6,7	21	23,6	22	24,7	36	40,4	13	14,6	13	14,6	23	25,8
Sim	15	25,0	13	21,7	6	10	20	33,3	16	26,7	27	45,0	10	16,7	10	16,7	19	31,7
*p*	0,111^*^		0,666^*^		0,546^**^		0,192^*^		0,789^*^		0,581^*^		0,733^*^		0,733^*^		0,43^**^	
Horas de sono diárias (n=146)																		
Menos 8 horas	20	20,0	23	23,0	8	8,0	33	33,0	30	30,0	44	44,0	18	18,0	16	16,0	34	34,0
8 ou mais horas	8	17,4	11	23,9	4	8,7		15,2	8	17,4	18	39,1	5	10,9	7	15,2	8	17,4
*p*	0,710		0,903		0,887		0,025		0,107		0,580		0,272		0,904		0,039	
Atividade física																		
Não	19	19,8	23	24,0	11	11,5	28	29,2	28	29,2	45	46,9	17	17,7	18	18,8	33	34,4
Sim	9	21,4	12	28,6	0	0,0	12	28,6	7	16,7	14	33,3	4	9,5	4	9,5	7	16,7
Às vezes	0	0	0	0	1	9,1	1	9,1	3	27,3	4	36,4	2	18,2	1	9,1	2	18,2
*p*	0,313^***^		0,351^***^		0,043^*^		0,292^***^		0,321^***^		0,182^***^		0,498^***^		0,162^***^		0,041^***^	
Índice de Massa Corporal^*^																		
Até 24,9 (baixo até normal)	5	13,5	8	21,6	2	5,4	5	13,5	10	27,0	16	43,2	7	18,9	4	10,8	9	24,3
25 a 29,9 (sobrepeso)	13	22,4	14	24,1	4	6,9	19	32,8	14	24,1	26	44,8	7	12,1	9	15,5	16	27,6
30 a 40 (obesidade)	10	18,5	13	24,1	6	11,1	17	31,5	14	25,9	21	38,9	9	16,7	10	18,5	17	31,5
*p*	0,555^*^		0,953^*^		0,307^***^		0,088^*^		0,948^*^		0,809		0,634^*^		0,607^*^		0,751^*^	
Relação cintura/quadril																		
Normal	9	26,5	8	23,5	2	5,9	8	23,5	7	20,6	17	50,0	7	20,6	6	17,6	8	23,5
Elevado	19	16,5	27	23,5	10	8,7	33	28,7	31	27	46	40,0	16	13,9	17	14,8	34	29,6
*p*	0,192^*^		0,995		0,735^**^		0,553		0,454		0,300^*^		0,344^*^		0,685		0,492^*^	

* Teste qui-quadrado;

** Teste exato de Fisher;

*** Teste qui-quadrado com correção.

Obteve-se associação estatística significativa entre a DME na região dos joelhos, relatada nos sete dias anteriores à entrevista, e os trabalhadores do SHL casados e/ou com companheiros (p=0,048) (Tabela 2).

A DME na região do pescoço, nos sete dias anteriores à entrevista, esteve associada estatisticamente à insatisfação com o número de pessoas na escala de trabalho (p=0,019) (Tabela 3).

Nos sete dias anteriores à entrevista, estiveram associados à DME no pescoço os trabalhadores do SHL que utilizam, às vezes, algum tipo de bebida alcoólica (p=0,048). Nas regiões dos ombros e na parte inferior das costas, esteve associada à prática de automedicação (p=0,026 e p=0,020, respectivamente); na região dos cotovelos, à não realização de atividade física (sedentarismo) (p=0,043); nos punhos e mãos, aos trabalhadores ex-tabagistas (fumei, mas parei) (p=0,015), bem como aos usuários de algum tipo de medicamento (p=0,004). Já nos membros inferiores, a dor na região dos joelhos associou-se ao uso ocasional de bebida alcoólica (p=0,004). A dor na região dos tornozelos e pés associou-se ao sedentarismo (p=0,041) e a dormir menos de oito horas de sono diárias (p=0,039).

## DISCUSSÃO

Evidenciou-se a prevalência de DME nos trabalhadores do SHL, nos sete dias anteriores à realização do questionário, na região lombar (42,3%), seguida pela região dos tornozelos e pés (28,2%), dos punhos ou mãos (27,5%), da parte superior das costas (25,5%) e dos ombros (23,5%). Esses acometimentos são indicadores preocupantes, pois retratam dores em período recente, podendo estar associada ao processo de trabalho de limpeza. Estudo com trabalhadores de limpeza terceirizados de uma faculdade pública identificou que 22,7% já sofreram afastamento por doenças osteomusculares. Ainda, 81,8% relataram sintomatologia de DME relacionada ao tipo de atividade desempenhada e às condições de trabalho^(15)^.

Segundo a Pesquisa Nacional de Saúde^(16)^, cerca de 34,3 milhões de brasileiros (21,6%) relataram dores crônicas na coluna, sendo que 2,5% da população possuem diagnóstico de distúrbios osteomusculares relacionados ao trabalho. Esses acometimentos podem estar relacionados à manutenção das posturas em pé por períodos prolongados e, consequentemente, sobrecarga da coluna vertebral. Ainda, quando associados à postura inadequada, ao levantamento manual de carga, flexão, torção e inclinação da coluna com movimentos repetitivos, podem resultar em sintomatologia dolorosa^(17)^.

Neste estudo, trabalhadores que realizam automedicação foram associados ao desenvolvimento de lombalgias (p=0,020) e DME na região dos ombros (p=0,026). Corroborando a hipótese dessa relação, evidencia-se a utilização de medicações analgésicas e anti-inflamatórias pelos trabalhadores do SHL como forma de atenuar os sintomas de dor.

A DME nos tornozelos e pés, o segundo segmento com maior acometimento, indica o ortostatismo prolongado como um fator importante a ser analisado. Isso porque a permanência da postura em pé, sem pausa, associada, muitas vezes, ao tipo de calçado utilizado e às superfícies rígidas, ocasiona o aumento da pressão dos músculos plantares e baixo limiar para os sintomas vasculares, resultando em quadros álgicos^(18)^. Dessa forma, prolongar a permanência nesta posição pode representar risco biomecânico aos trabalhadores do SHL, expondo estruturas musculares e de articulações para sustentação corporal^(18)^. Como estratégia para minimização da ocorrência de DME nos tornozelos e pés, recomenda-se a intercalação das atividades que exigem o ortostatismo prolongado, como a de higiene e limpeza de ambientes, com a postura sentada ou andando, o que permite alternância de diferentes grupos musculares^(19)^.

A associação entre a DME nos tornozelos e pés e o sedentarismo (p=0,041) aproxima-se do fato de os trabalhadores que não realizam atividade física regularmente possuírem preparo físico enfraquecido para as atividades com alta exigência. Nesse sentido, a inatividade física pode levar à ocorrência de atrofias musculares e de tecidos conjuntivos de suporte. Além disso, o sedentarismo relaciona-se ao aumento da rigidez dos tendões, fáscias, ligamentos e músculos^(20)^. Para tanto, investigações apontam que a atividade física orientada é efetiva para manter e restabelecer a capacidade para o trabalho, aumentando a oxigenação em nível celular, diminuindo o estresse e melhorando a autoestima^(20,21)^.

Já a DME nos punhos ou mãos, terceiro segmento corporal acometido, ocorre devido ao processo de trabalho do SHL ser dotado da utilização constante dos membros superiores, com atividades de manuseio das mãos na limpeza e na desinfecção de superfícies, bancadas e equipamentos^(21)^. Ademais, o esforço exigido pelos punhos e mãos também é modulado pela qualidade e pelo tipo de equipamento (extensor do rodo, esfregão e máquina lavadora).

Entre os fatores que podem influenciar a ocorrência das lesões durante o uso das mãos, encontram-se o peso e o tipo da carga^(21)^. Quando o formato da carga/equipamento se aproxima da anatomia das mãos, é proporcionado maior contato com o objeto, permitindo maior firmeza de preensão. Nesse caso, uma quantidade menor de força é utilizada, facilitando o processo. Ao mesmo tempo, um objeto maior necessita de mais força para mantê-lo e um número maior de segmentos corporais são recrutados para estabilizá-lo^(22)^.

Os tabagistas e ex-tabagistas, juntos, apresentaram maior frequência de DME na coluna lombar (81,9%), tornozelos e pés (62,7%), e quadril e coxas (61,8%). Os indícios prováveis dessa associação podem estar relacionados à alteração de pH e à nutrição dos discos intervertebrais pelos componentes do cigarro, predispondo às herniações. Ainda, há conexão do uso do tabaco com a diminuição da resistência dos músculos estabilizadores da coluna lombar, predispondo à dor. É possível que a nicotina afete o sistema nervoso central e ocasione vasoconstrição e diminuição da oxigenação celular, interferindo na percepção da dor^(23)^.

O quarto segmento com maior acometimento por DME foi a parte superior das costas/coluna torácica (25,5%). Esta é a segunda região da coluna vertebral, posicionada entre a coluna cervical e a lombar. A postura de flexão e rotação da coluna vertebral exigida no trabalho de limpeza, associada ao levantamento de peso (sacos de lixos), configura-se maior agravante em relação à DME na coluna torácica. Similarmente ao serviço de varrição urbana, a presença de sintomas nessa região diminui a capacidade de trabalho^(24)^.

Outro fator de risco importante é a realização de movimentos repetitivos rápidos, como a limpeza debaixo dos leitos hospitalares. Autores^(23,24)^ relatam que as posturas de trabalho desconfortáveis, limitadas, assimétricas, repetidas e/ou prolongadas, bem como os movimentos extremos e/ou repetitivos e emprego de força excessiva, podem causar sobrecarga nos tecidos e exceder seus limites de estresse, causando lesões teciduais devido à sobrecarga nas estruturas musculoesqueléticas, principalmente da coluna vertebral.

No que tange à DME na região dos ombros, há a permanência dos trabalhadores do SHL, por extensos períodos, com a postura de braços elevados, sem apoio. Este achado também pode ser justificado pela alta taxa de repetição de movimentos, bem como pela biomecânica gestual, característica da função de varrer, além do manuseio de carga, elevação de membros superiores e colocação do lixo no carrinho^(24)^.

Não foi evidenciada associação significativa entre obesidade e DME nos segmentos corporais avaliados. Porém, dos trabalhadores do SHL, 38,9% apresentavam-se com excesso de peso corporal (IMC de 25 a 29,9 kg/m^2^), e 36,2% possuíam obesidade graus I, II e III. Torna-se importante considerá-la uma comorbidade e um fator de risco considerável no acometimento por DME. Embora os trabalhadores realizem atividades que ocasionam gasto de energia, aumento do batimento cardíaco e relatem possuir tempo para o lazer, essas não são suficientes para evitar o sobrepeso. Logo, faz-se necessário o incentivo às práticas regulares de atividades físicas, a fim de melhorar a força, a flexibilidade e a resistência muscular localizada, promovendo a qualidade de vida e o aumento da capacidade para o trabalho^(25)^.

Este estudo permitiu inferir que a exposição aos fatores ergonômicos pode ocasionar sobrecarga nos tecidos e exceder seus limites de estresse, causando lesões teciduais em virtude de esforços inadequados e sobrecarga nas estruturas musculoesqueléticas, principalmente da coluna vertebral. Com isso, há a ocorrência de DME, em que o impacto é pronunciado quando ocorre a combinação de dois ou mais desses fatores de risco em uma única atividade.

### Limitações do estudo

Considera-se como limitação desta investigação não ser possível determinar a relação de causa e efeito entre exposição e desfecho, condição relacionada a investigações transversais.

### Contribuições para as áreas da enfermagem e saúde

Além de buscar a reflexão e discussão sobre os fatores associados à exposição à DME presentes no trabalho de limpeza, o conhecimento científico é relacionado à saúde dos trabalhadores de apoio, como o SHL. Os resultados podem ser úteis para a compreensão dos fatores de risco ergonômicos existentes no trabalho de limpeza e quais estratégias podem auxiliar para a minimização da ocorrência de DME e, sobretudo, facilitar o processo de transformação e promoção de ambientes saudáveis de trabalho.

## CONCLUSÕES

Os trabalhadores do SHL investigados realizavam suas atividades laborais na presença de sintomas musculoesqueléticos consideráveis, principalmente as lombalgias. Os fatores associados à ocorrência de DME estão relacionados às variáveis laborais e a hábitos de saúde e vida como: insatisfação e o número de pessoas na escala de trabalho; utilização esporádica de algum tipo de bebida alcóolica e dor no pescoço; automedicação e dor na parte inferior das costas e nos ombros; sedentarismo e dor nos cotovelos; ex-tabagismo, uso de medicamento e dor nos punhos ou mãos; uso ocasional de bebida alcoólica e dor nos joelhos; e sedentarismo, horas de sono diárias e dor nos tornozelos e pés.

Recomendamos que a gestão das instituições, nas quais há a atuação do SHL, protagonize ações para reduzir os riscos ergonômicos e os resultados negativos do trabalho, promova seu devido reconhecimento e invista em manutenção de programas de capacitação e aprimoramento do processo de trabalho, com vistas à minimização da exposição aos riscos ergonômicos e à DME. Somado a isso, há a necessidade de intervir nas condições laborais da limpeza hospitalar, a fim de diminuir as exposições à DME e promover as condições para o trabalho saudável. Um programa de educação em saúde e recomendações de melhorias nos processos de trabalho, direcionados aos trabalhadores e à gestão do SHL, podem minimizar a exposição à DME. Por fim, sugerimos o desenvolvimento de pesquisas com delineamentos longitudinais e experimentais, incluindo amostras maiores e outros cenários hospitalares.
